# Impacts of a novel defensive symbiosis on the nematode host microbiome

**DOI:** 10.1186/s12866-020-01845-0

**Published:** 2020-06-15

**Authors:** Dylan Dahan, Gail M. Preston, Jordan Sealey, Kayla C. King

**Affiliations:** 1 Department of Zoology, University of Oxford, Oxford, OX1 3SZ UK; 2 Department of Microbiology and Immunology, Stanford University School of Medicine, Stanford, CA 94305-5124 USA; 3 Department of Plant Sciences, University of Oxford, Oxford, OX1 3RB UK; 4 School of Cellular and Molecular Medicine, University of Bristol, Bristol, BS8 1TD UK

**Keywords:** *Caenorhabditis elegans*, Defensive symbiosis, *Enterococcus faecalis*, Evolution, Microbiome, Pathogen, Protection

## Abstract

**Background:**

Bacteria adapted to live within animals can protect their hosts against harmful infections. Beyond antagonism with pathogens, a ‘defensive’ bacterial symbiont could engage in additional interactions with other colonizing micro-organisms. A single bacterium might thus have cascading ecological impacts on the whole microbiome that are rarely investigated. Here, we assess the role of a defensive symbiont as a driver of host-associated microbiota composition by using a bacterial species (*Enterococcus faecalis*) that was previously experimentally adapted to a nematode host model (*Caenorhabditis elegans*).

**Results:**

An analysis of 16S rRNA data from *C. elegans* exposed to *E. faecalis* and subsequently reared in soil, reveal that symbiont adaptation to host environment or its protective potential had minimal impact on microbiota diversity. Whilst the abundance of *Pseudomonas* was higher in the microbiota of hosts with protective *E.faecalis* (and another protective species tested), a few other genera – including *Serratia* and *Salinispora –* were less abundant in hosts colonized by all *E. faecalis* strains. In addition, the protective effect of *E. faecalis* against virulent *Staphylococcus aureus* pathogens was maintained despite multi-species interactions within the microbiota.

**Conclusions:**

Our results reveal the degree to which a new, evolving symbiont can colonise and maintain pathogen-resistance with minimal disruption to host microbiota diversity.

## Background

Animals can harbor a diversity of microbes. Many of these micro-organisms can be beneficial and protect their hosts against pathogen infection [1, 2]. These defensive microbial symbionts can be important in determining infection outcomes across natural host populations [3, 4], and for hosts in agricultural or biomedical contexts [5–8]. It has also been suggested that defensive microbial symbionts might help to prevent the transmission of devastating vector-borne diseases to humans [9].

Hosts are protected when these defensive symbionts, for example, block the growth or establishment of pathogens. These symbionts can suppress invading parasites and pathogens directly via toxin production [10] or offer protection through influence on their host immune systems and microbiomes [5, 11]. These protective mechanisms might also have cascading ecological impacts on the whole microbiome within the host, particularly if the mechanism is not highly specific (e.g., bacteriocin secretion), but effective against a broad-spectrum of pathogen isolates or species [12, 13]. With competition thought to dominate species interactions within the microbiome [14], protectors could exclude casual colonizers or less competitive symbionts from the microbiota. By interacting with other species or shifting microbiota composition, symbionts might have negative effects on hosts by inadvertently increasing the infection load of pathogens [15, 16]. The protective phenotype conferred to the host could be diminished or lost. The degree to which individual symbionts can shape the composition of other constituents of host microbiota, such as core (i.e., essential microbes conserved in the majority of a species microbiomes) members, is unclear. The microbiota can have a huge impact on host biology and health [17]. Thus, symbiont-mediated shifts in microbial communities could ultimately cause differences in in host phenotypes within and across populations.

Here, we test the impact of a bacterial symbiont on host microbiome structure, throughout its in vivo evolution. We used a bacterium (*Enterococcus faecalis*) that forms a novel interaction with the animal host, *Caenorhabditis elegans*, and was experimentally evolved within host populations [10]. The selected *E. faecalis* populations vary in their ability to directly suppress infection by a virulent pathogen (*Staphylococcus aureus*) via reactive oxygen species [10]. *C. elegans* nematodes have a defined core microbiota [18–20] acquired in their natural habitats [20], and the *E. faecalis* strains used herein allow us to test for impacts of symbiont presence, protective ability, and evolutionary history on the host microbiome. We introduced symbiont-colonized nematodes to microbial communities in compost (to mimic natural microbiota colonization), allowed a microbiome to assemble inside the worm [following 19], and then conducted 16S rRNA sequencing. Since *E. faecalis* can suppress *S. aureus* colonization using an antimicrobial mechanism that is not species specific (i.e., reactive oxygen species production), we predicted that other members of the nematode microbiome would also be inhibited. We further hypothesized that the protective effects of *E. faecalis* would remain in microbiome-colonised hosts, but be less effective overall. It has been previously shown that fitness constraints from multiple interactions in polymicrobial communities can dilute phenotypes normally observed in reduced systems [21]. However, the minimal shifts in microbiome composition we found suggests that novel, rapidly evolving symbionts can be maintained and remain effective without major disruption to the native microbiome.

## Results

We allowed *C. elegans* to be colonized early in life with bacterial symbionts, followed with microbiota assembly in compost, and then we conducted 16S rRNA sequencing of nematodes to examine microbiota composition. Symbiont treatments included three populations of *E. faecalis* and an additional bacteria species, *Pseudomonas mendocina*. The evolved *E. faecalis* population conferring enhanced protection (*E. faecalis* P), evolved *E. faecalis* not conferring enhanced protection (*E. faecalis* NP), and the ancestral *E. faecalis* (*E. faecalis* Anc) correspond to a randomly-selected replicate population from CCE *E. faecalis,* SE *E. faecalis,* and ancestral *E. faecalis* populations, respectively, in King et al. (2016) [10]. These populations have different genetic compositions, and *E. faecalis* P contains non-synonymous SNPs in genes putatively associated with superoxide production, a mechanism for pathogen suppression [10]. Increased superoxide production by *E. faecalis* in this system does not differentially affect host survival [22]. We used *P. mendocina* since it has previously been shown to limit colonization of *Pseudomonas aeruginosa* pathogens by inducing nematode immune responses [23]. We reasoned this bacterial species might also shape nematode microbiota if hosts were more likely to resist colonization via immune mechanisms. We sought to understand how early colonization with symbionts can shape microbiome diversity, since deviations from diversity due to early colonization can link with adverse outcomes for health host [19, 24].

### Colonization by *E. faecalis* did not change microbiome diversity

We retained on average 46,317 16S rRNA reads per microbiome across 75 *C. elegans* microbiome samples after quality filtering and preprocessing. Reads were processed into unique amplicon sequence variants (ASVs), where each ASV represents a unique 16S rRNA read, or a rough proxy for a microbial species. Early colonization by symbionts had a significant effect on observed ASVs (*F*_(4,39)_ = 3.84, *P* < 0.01) and Chao 1 diversity (*F*_(4,39)_ = 3.67 *P* = 0.012) but not Shannon diversity (*F*_(4,39)_ = 0.478, *P* = 0.75) (Fig. S1; Tables S1–S4), likely indicating major differences were driven by ASV richness and the abundance of rare ASVs. *Post-hoc* analyses suggested that significant differences were driven by low ASV diversity in samples exposed to *P. mendocina* and not evolved *E. faecalis* strains (Tables S2, S3). Colonization by *E. faecalis* strains had no significant effects on microbiota alpha diversity within nematode hosts.

**Fig. 1:**
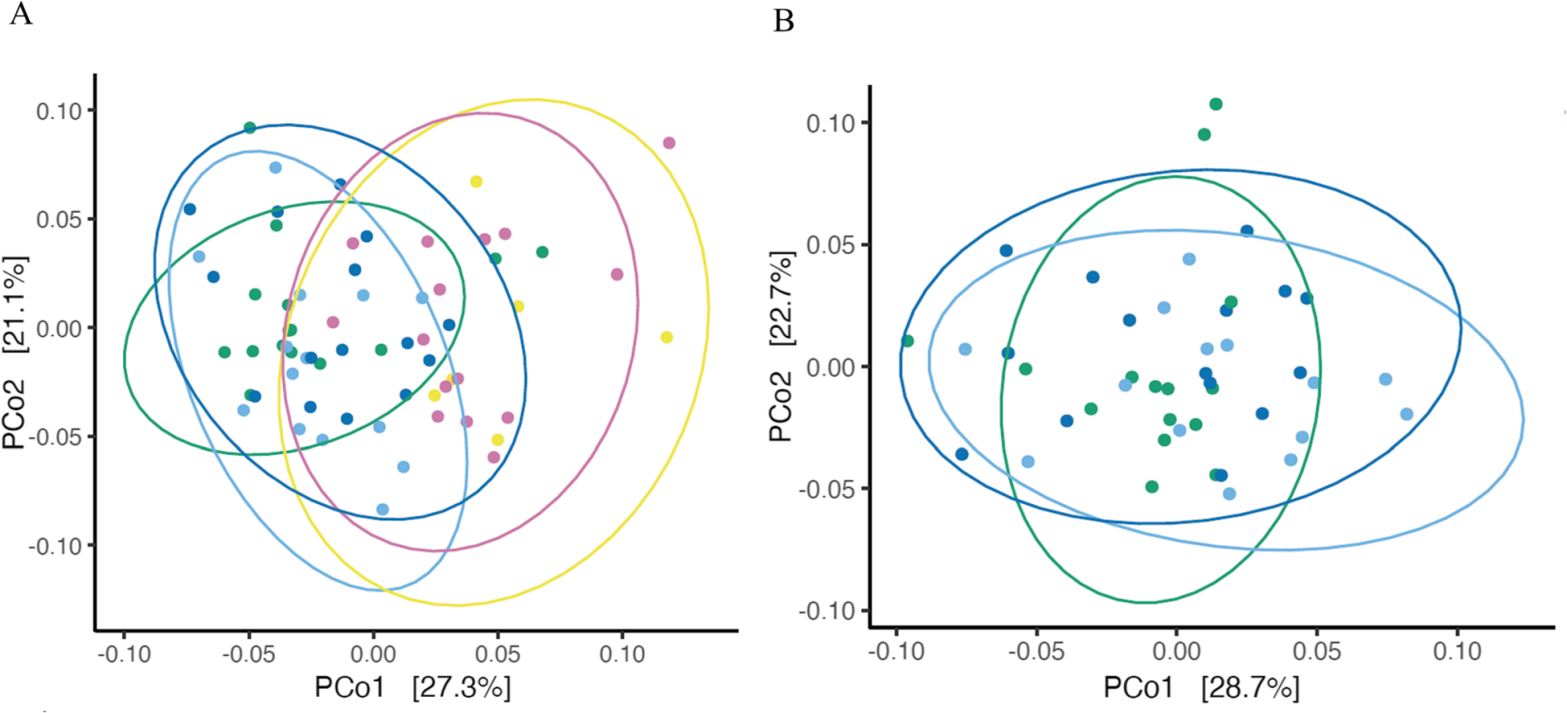
Principal coordinate analyses (PCoA) on weighted UniFrac scores of *C. elegans* microbiota. **a.** PCoA on weighted UniFrac scores by symbiont treatment. Treatment was as a significant predictor of ecosystem distance. **b.** PCoA on weighted UniFrac scores comparing microbiota from *E. faecalis* strain treatments. Early colonization by *E. faecalis* strains was not a significant predictor of ecosystem distance. Ellipses are drawn at 95% confidence intervals. Yellow, OP50 food; Green, ancestral *E. faecalis*; Dark blue, NP, *E. faecalis* no enhanced protection; Light blue, *E. faecalis* enhanced protection; Purple, *P. mendocina*

In our beta diversity analyses, the first two axes explained more than 50% of sample variance (Fig. 1; PCo1 = 28.7% and PCo2 = 22.7%) and a marginal batch effect remained after removing it (ANOSIM; R^2^ = 0.083; *P* = 0.01; see “Methods”). Colonization treatment was a small but significant predictor of discernably clustering *C. elegans* microbiota diversity (Fig. 1a; ANOSIM; R^2^ = 0.201; *P* = 0.001). Amongst *E. faecalis* treatments, there were no significant differences in beta diversity (Fig. 1b; ANOSIM; R^2^ = 0.01; *P* = 0.34). This result reveals that the observed differences in beta diversity were driven by differences between symbiont species (*E.faecalis* vs. *P. mendocina*) and not by differences between *E. faecalis* strains.

### Microbiota differential abundance influenced by symbionts

**Fig. 2:**
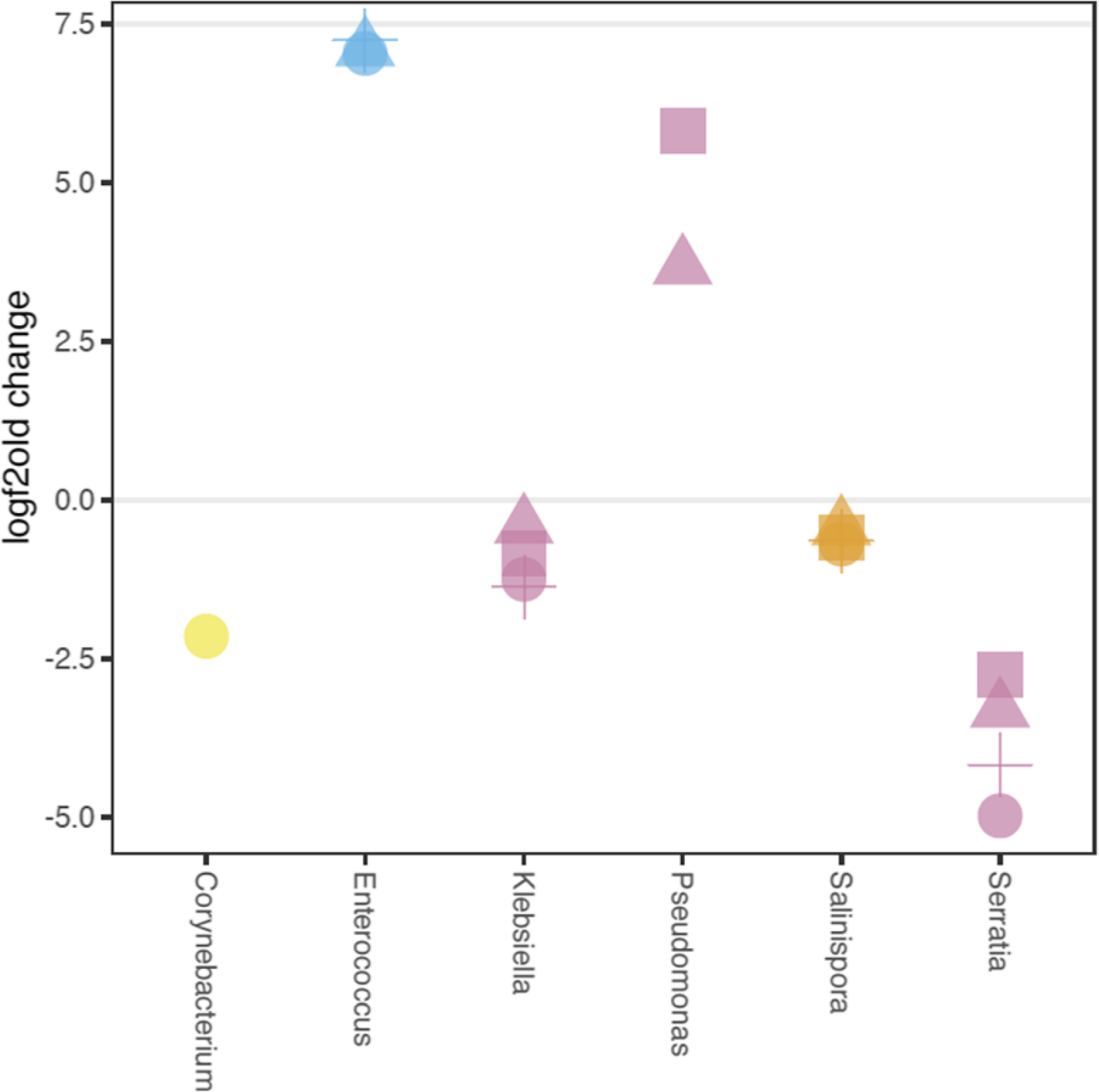
Microbes that significantly differed in abundance with symbiont treatments. Log2fold change of significantly differentially abundant ASVs identified by comparing microbiota of *C. elegans* colonized by different symbionts relative to those only given food. Circle, ancestral *E. faecalis;* triangle, *E. faecalis* enhanced protection; cross, *E. faecalis*, no enhanced protection; square, *P. mendocina.* Yellow, Actinobacteria; blue, Bacilli; orange, betaproteobacteria; purple: gammaproteobacteria

Early exposure to a symbiont can negatively shape host-microbiota by subsequently increasing pathogen colonization [25]. We sought to understand if early exposure to *E. faecalis* P impacted the normal colonization of subsequent microbiota members. We measured how the different *E. faecalis* strains influenced significantly changing taxa abundance. Comparing the microbiota in hosts colonized by *E. faecalis* strains and *P. mendocina* relative to the non-symbiont control (i.e., OP50), we observed that all three *E. faecalis* strains increased the abundance of an ASV identified as *Enterococcus* by an average of 7.13 log2fold (Fig. 2; ANCOM; adj-*P* < 0.01). We hypothesize that the *E. faecalis* strains to which we exposed hosts were at increased abundance, but lack of strain-level resolution sequencing limits our conclusions. In this case, *Enterococcus* was from the environment compost communities only. We found that *P. mendocina* and *E. faecalis* P exposures significantly increased abundance of *Pseudomonas* by an average of 5.82 log2fold (Fig. 2; ANCOM; adj-*P* < 0.01). We also observed a decrease in abundance of *Serratia, Klebsiella* and *Salinispora* in the *E. faecalis* P, NP, and Anc treatments (Fig. 2; ANCOM; adj-P < 0.01). Interestingly, the *E. faecalis* Anc exposures uniquely decreased the abundance of *Corynebacterium* (Fig. 2; ANCOM; adj-P < 0.01). We also measured the differential abundance of microbiota among *E. faecalis* strain exposures but did not find any significant differences.

### Protection by *E. faecalis* maintained amidst microbiota

**Fig. 3:**
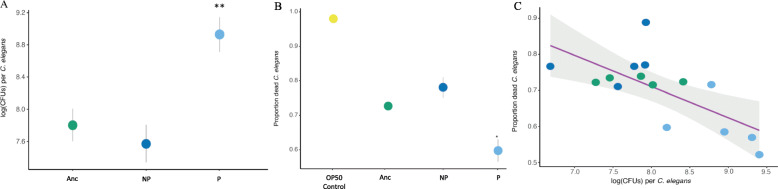
*E. faecalis* conferring enhanced protection colonizes hosts at higher density and reduces pathogen-induced host mortality in a microbiome context. **a.** Within-host colony-forming units of evolved and ancestral *E. faecalis* symbiont (log_e_CFUs presented). **b.***C. elegans* mortality after symbiont, compost, and pathogen exposures. **c.** Correlation between *E. faecalis* colonization density pathogen-induced mortality in hosts with a microbiota. *n* = 5 populations per treatment. Error bars = ± s.e. Yellow, OP50 food; Green, Anc, ancestral *E. faecalis*; Dark blue, NP, *E. faecalis* no enhanced protection; Light blue, P, *E. faecalis* enhanced protection

We examined whether within-host adaptation by *E. faecalis* P resulted in increased within-host density and protection persistence in the nematode-microbiome system. Nematodes were colonized by, on average, 3.43x more *E. faecalis* P colony forming units (cfus) (mean = 8201 cfus; s.e. = 1540), than *E. faecalis* Anc (mean = 2664; s.e. = 543) and *E. faecalis* NP (mean = 2125; s.e. = 365) before pathogen infection, a finding that was significant (Fig. 3a; CCE to Anc = one-tailed t-test t = 3.39, df = 4.98, adj-*P* = 0.015; CCE to SE = one-tailed t-test, t = 3.83, df = 4.45, adj-P = 0.015).

Symbiont-conferred phenotypes can be diluted in natural contexts. We therefore investigated protection persistence of *E. faecalis* P in hosts with a microbiota, and found that protection was maintained (Fig. 3b). Early exposure to *E. faecalis* P, resulted in ~ 16.5% lower mortality during infection with *Staphylococcus aureus* compared to that with *E. faecalis* Anc or *E. faecalis* NP (Fig. 3b; Wilcoxon test; one-tailed; adj-Ps = 0.016). Our results further indicate that initial colonization by the *E. faecalis* symbiont was a significant predictor of reduced mortality due to pathogen infection (Fig. 3c; Pearson’s; R = − 0.775; t = − 4.42; df = 13; *p* < 0.01). We found that *E. faecalis* colonization did not correlate with the relative abundance of *Enterococcus* taxa (Pearson’s, R = − 0.642; t = − 0.839; df = 1; *p* = 0.556; Fig. S2). Moreover, the relative abundance of *Enterococcus* after microbiome assembly did not predict decreased *S. aureus*-induced mortality (Pearson’s; R = 0.788; t = 1.28; df = 1; *p* = 0.422; Fig. S3). These results suggest that *E. faecalis* strain type and early colonization abundance, but not *Enterococcus* relative abundance in the greater microbiota, was important for protection against *S. aureus* pathogens.

## Discussion

Defensive bacteria have been found in the microbiota of a diversity of animal species [26]. We hypothesized that competition between defensive symbionts and other colonizing micro-organisms would impact microbiome diversity and structure, and we examined changes caused by ancestral and evolved strains. Here, we found that early colonization by an experimentally-adapted protective symbiont did not have significant effects on *C. elegans* microbiome diversity. The abundances of three microbiome components were reduced, a pattern consistent across nematodes colonized by all strains of the symbiont species tested.

Symbionts can have synergistic or antagonistic effects on others, effectively shifting symbiont services and costs [11, 25]. We found that nematode host microbiota diversity was not greatly affected by the early colonization of *E. faecalis*, regardless of evolutionary history or protective ability. Out of all early exposure treatments, only *P. mendocina* significantly decreased microbiome alpha diversity. This result could suggest that symbionts that protect by launching the host immune response might cause more substantive shifts in microbiome structure, particularly if host control plays a key role in maintaining the microbiome [27]. In human systems, low microbiome diversity has been associated with adverse health outcomes [24], but this link remains largely untested in wild animal systems (although see [28]). Across all strains, *E. faecalis* was found to minimally drive beta-diversity and therefore microbiome assembly. Convergence towards a “normal” microbiome regardless of early colonization is common in other hosts [29].

The minimal effects we did notice included an increased abundance of the core microbe *Pseudomonas* in the *E. faecalis* P treatment and decreased abundance of *Serratia, Klebsiella* and *Salinispora* in all treatments with *E. faecalis* strains. The results could be linked to microbe-microbe interactions or a host-mediated response, such as increased ROS inducing higher expression of genes associated with antagonism to other bacteria or with host colonization [30, 31]. The loss of *Serratia* was the most significant. Reductions in the abundance of this genus from the microbiome might thus have consequences for nematode longevity and fitness not investigated in this study. Some strains and species of *Serratia* can be beneficial or pathogenic to *Caenorhabditis* [32, 33], and pathogenic *S. marcescens* can drive nematode evolution and mode of reproduction [34].

The pattern of *E. faecalis* protective ability amongst strains was maintained in nematodes with a natural microbiome. However, the strength of defence was reduced relative to mono-colonization of this symbiont [10], suggesting a dilution effect caused by the microbiome. This finding is consistent with other studies showing that fitness constraints imparted by diverse interactions in polymicrobial communities can change [35] and dilute [11, 36] phenotypes normally observed in reduced systems. Indeed, symbiont strains with distinct functions can persist in hosts [37], allowing for additive symbiont genetic and phenotypic diversity. *E. faecalis* P’s sustained protection amongst a more natural-like setting is promising for the application of probiotic microbes to hosts with a diverse microbiota. In some wild species-rich communities, microbe-mediated host protection can be diminished [21].

## Conclusions

An *E. faecalis* strain experimentally evolved to protect *C. elegans* against infection had minimal impact on the host microbiome. This finding supports the idea that we can expand methods for yielding beneficial components of the microbiome beyond current methods to include genetic engineering of probiotics [8] or using experimental evolution. For example, defensive *E. faecalis* did not alter the microbiota diversity or core microbiome members to a significant degree, yet still provided strong protection against a deadly pathogen. These results highlight that similarly-derived bacteria could be robust therapeutics, offering beneficial effects without disrupting microbiome health. However, it must be re-emphasized that the protective strains studied herein were evolved in the absence of a complex microbial community with the animal host. The community context can alter the evolutionary outcomes of individual microbe species [38, 39]. Future research could test whether microbe-mediated protection can be enhanced via evolution in hosts with a resident microbiota.

## Methods

### Tripartite host-symbiont-pathogen system

We used *C. elegans* Bristol N2 strain obtained from *Caenorhabditis* Genetic Center. They were maintained using standard procedures (www.wormbook.org), and fed *Escherichia coli* OP50 on Nematode Growth Medium (NGM). Bacterial *E. faecalis* strains were *E. faecalis* OG1RF (aka Anc) [40], a strain from the human gastrointestinal tract, and randomly selected colonies of *E. faecalis* SE (NP herein) and *E. faecalis* CCE (P herein) from previously evolved populations [10]. The *Pseudomonas mendocina* strain used was isolated from the soil by M. Shapira and was found to be capable of colonizing nematodes as part of their microbiome [23]. The *S. aureus* strain used was MSSA476 [41], a disease-causing pathogen which can cause harm to worm hosts during infection [10].

### Experimental approach

**Fig. 4:**
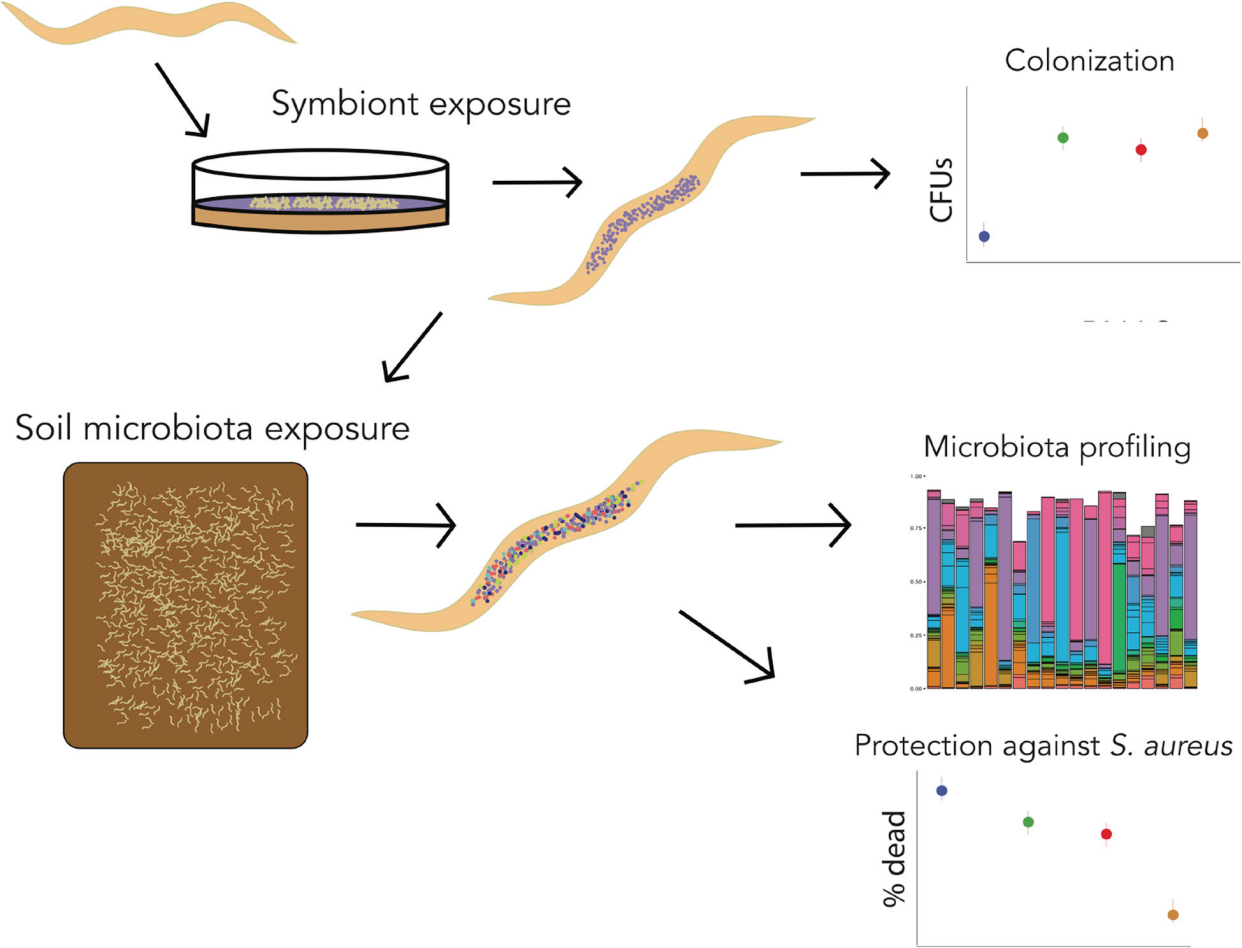
Graphical assay abstract. *C. elegans* exposed to different defensive symbiont strains underwent 16S rRNA analysis and phenotypic assays. Microbiota profiling as well as symbiont colonization enumeration and protection persistence were assessed

We provide a graphical abstract of assays related to our results in Fig. 4.

#### *C. elegans* exposures to food and bacteria

These nematodes are easily reared in a gnotobiotic setting allowing for controlled assembly of microbes in their gut [10, 42]. Culturing of and *C. elegans* exposure to *E. faecalis* Anc, *E. faecalis* NP, or *E. faecalis* P were the same as in King et al. (2016) [10], with a different washing procedure that was described by Ford et al. (2016) [43]. Washing removed the majority of externally adhering bacteria and included removing cutaneous microbes by washing nematodes three times with M9 buffer over a filter tip and spinning at 800 g. For all experiments, eggs were obtained from gravid nematodes by bleaching. Approximately 1000 nematodes were exposed as L1s to OP50 on NGM at 20 °C and allowed to develop for 24 h. After being washed, nematodes were transferred to the food control OP50 or one of four symbiont treatment exposures – *E. faecalis* strains (Anc, NP, or P), or *P. mendocina* - at 25 °C for 24 h. All bacteria were cultured overnight in *Luria-Bertani* broth (LB) (OP50 and *P. mendocina*) or Todd-Hewitt Broth (*E. faecalis* and *S. aureus*) before being plated on NGM (OP50, 100 μL) or Tryptone Soy Broth Agar (TSA; *E. faecalis*, *P. mendocina*, *S. aureus*; all at 60 μL) and cultured for 24 h at 30 °C. Culture and exposure procedures were consistent across all assays (RNA extraction, soil exposure, gut accumulation, and protection persistence), with differences only in replicates, batch numbers and treatment exposures, referred to as the standard experimental exposure.

#### Compost preparation

The procedure for compost preparation followed Berg et al. 2016 [19]. Overripe bananas were supplemented to Westland Multi-Purpose Compost with added John Innes (Westland Horticulture; Dungannon, UK) to enrich microbiota via carbohydrates. They were left to compost at 20 °C for 5 days before the mixture was disrupted and washed to create a microbial extract. To create the microbial extract, we added 2 mL M9 to 5 g compost in a 50 mL conical tube, vortexed vigorously for 60 s, transferred a 10 mL aliquot to a 15 mL conical tube and centrifuged the mixture for one minute at 300 g, and created a glycerol stock (25%) of the wash that was immediately stored at − 80 °C. To reconstitute compost with microbes prior to worm addition, 5 g of autoclaved compost was supplemented with 1 mL microbial wash and incubated for 48 h at 25 °C.

#### Worm compost exposure and harvesting

Five replicates of each treatment repeated over three replicate batches were used for compost exposures. Following the standard treatment exposure, nematodes were repeatedly washed and transferred to microbial enriched soil for 24 h, after which ~ 700 nematodes were harvested over 2 h using a Baermann funnel lined with tissue paper [as in 19], then washed again and immediately stored at − 80 °C until DNA extractions.

#### DNA extractions

Genomic DNA was isolated from compost exposed nematodes (~ 700) or soil (0.25 g) using the MO BIO PowerSoil DNA Isolation Kit (12,888; MO BIO Laboratories; Carlsbad, CA, USA), with slight adjustments. For homogenization and cell lysis, we attached the MO BIO kit’s PowerBead Tubes to the Benchmark Scientific BeadBlaster Homogenizer (D1030-E; Benchmark Scientific; South Plainfield, NJ, USA) and homogenized and lysed cells for 60 s at 2800 rpm. Final gDNA was released from the silica membrane using 40 μL sterile, nuclease-free water (Promega; Madison, WI, USA).

#### 16S rRNA library preparation

The 16S rRNA V4 region was amplified from the worm microbiome gDNA using the 515F Golay-barcoded primers and 806R, primers revised by Apprill et al. and developed by Caporaso et al. and listed on the Earth Microbiome Project (EMP) 16S protocol site (http://www.earthmicrobiome.org/emp-standard-protocols/16s/). Samples were prepared in accordance with the standard EMP 16S rRNA protocol. 25 μL polymerase-chain reactions contained 10 μL Platinum Hot Start MM (2X) (company), 11 μL nuclease-free water, 1 μL of each forward and reverse primer (0.20 uM final concentrations), and 2 μL Genomic DNA (gDNA) template. No-template controls contained nuclease-free water in lieu of gDNA. Reactions were held at 94 **°**C for 3 min to denature the DNA, and amplification took place for 35 cycles at 94 **°**C for 45 s, 50 **°**C for 60s and, 72 **°**C for 90s. The cycles were followed by a hold at 72 **°**C for 10 min. Amplicons were visualized on a 1.5% agarose gel. gDNA was quantified using the Qubit 2.0 (Thermofisher, Bartlesville, OK) and amplicons were pooled at equimolar ratios (~ 240 ng per sample). The combined amplicon pool was then cleaned using the Qiagen PCR Purification Kit (Qiagen, Germantown, MD). The multiplexed library was quality checked and sequenced with the MiSeq 2x250nt PE v2 protocol at the W.M. Keck Center for Comparative and Functional Genomics (University of Illinois at Urbana-Champaign; Urbana, IL, USA).

#### Gut accumulation enumeration and protection persistence

Five replicates of each treatment from the same batch were used for gut accumulation enumeration and protection persistence assays. Following the standard treatment exposure, nematodes were repeatedly washed and then either transferred to microcentrifuge tubes containing ten 1 mm zirconia/silica beads in 50 μL M9, for the gut accumulation enumeration, or advanced to soil exposures for the protection persistence assay. The nematodes were homogenized and bacteria were released using the Benchmark Scientific BeadBlaster Homogenizer (D1030-E; Benchmark Scientific; South Plainfield, NJ, USA) for 45 s at 2800 rpm. Dilution series of the mixture were plated on TSA and cfus were enumerated after incubating at 30 **°**C for 24 h. For the protection persistence assay nematodes were transferred to plates with *S. aureus* and exposed for 24 h at 25 °C. After exposure, we calculated mortality by counting alive and dead nematodes.

### 16S rRNA bioinformatic processing and analyses

PhiX sequences were first removed from my library using Bowtie2 by mapping my reads against an index built from a phiX genome (found at support.illumina.com/sequencing/sequencing_software/igenome.html). Demultiplexed, paired-end fastq files were then processed in R (3.4.0) using DADA2 as previously described [44]. In short, this included filtering and trimming, error rate estimation, dereplication of reads into unique sequences, and ribosomal variant inference. We then merged paired-end reads, constructed amplicon sequence variant (ASV) table (sample x sequence abundance matrix), and removed chimeras. We also used DADA2’s native implementation of the Ribosomal Database Project (RDP) naïve Bayesian classifier trained against the GreenGenes 13.8 release reference fasta (https://zenodo.org/record/158955#.WQsM81Pyu2w) to classify ASVs taxonomically. For DADA2 and phyloseq processing we provide a reproducible R Markdown file (Supplementary File 1).

We described early exposure to symbionts effects on subsequent microbiota assembly and diversity using both within (alpha) and between (beta) sample diversity measurements. Observed ASVs indicates the number of ASVs per sample, the Shannon metric is an equal weighted metric for species richness and evenness, and the Chao 1 index is a metric weighted towards rare ASVs that also incorporates richness and evenness.

We created visualizations and conducted statistical analyses on the ASV table in R (3.4.0). To calculate alpha diversity measurements of observed ASVs, Shannon’s index and Chao 1, we used phyloseq’s (1.16.2) [45] estimate_richness function. Phyloseq was also used to perform ordinations, using principle coordinate analysis on UniFrac distance scores [46]. To perform differential abundances analyses, we used the ANCOM package [47]. Other R packages used include: ggplot2, for visualizing data and making Figs. (2.0.0); Rcpp for C++ parallelization in R; optparse (1.3.2.) to parse command line options; stats (3.2.3) to conduct statistics; and data.Table (1.9.6) to handle data frames. For our 16S rRNA analyses we have also provided an R markdown file outlining a fully reproducible workflow (Supplementary File 1).

### Statistical analysis

For all analyses we used R (v3.5.3). For all tests, we report exact n-values in figure legends. We also provide complete ANOVA tables, including F-values and degrees of freedom, as supplementary tables. For t-tests and Wilcoxon tests, we provide *P*-values, t-values and degrees of freedom in results.

Our microbiome samples were prepared in three independent batches, with 5 biological replicate per treatment in each batch, yielding a total of 75 *C. elegans* microbiome samples. After pre-processing, we retained 65 *C. elegans* population microbiome samples. For taxa, pre-processing included removing taxa from samples found in non-template controls, and removing taxa not observed at least once in 20% of samples. We corrected for a batch effect in beta diversity and differential taxa abundance analyses using a variance stabilizing transformation, which normalizes taxa count data based on depth factor and produces a matrix with values that are homoscedastic. We corrected for a batch effect in alpha diversity measurements by testing for batch as a significant predictor, then pruning batch three, which accounted for the majority of outliers.

To examine whether the different symbiont exposures affected microbiota alpha diversity, we used Analysis of Variance (ANOVA) tests with Tukey’s honest significant difference (HSD) for post-hoc comparisons. After rarefying, we retained 45 *C. elegans* microbiome samples for alpha diversity tests. We report exact n-values in figure legends and complete ANOVA tables, with F values and degrees of freedom.

To calculate beta diversity we first built a distance matrix based on samples’ weighted UniFrac scores [46], and performed principal coordinate analysis on the distance matrix. To test whether the symbiont exposures affected microbiota beta diversity, we used Analysis of Similarity (ANOSIM) tests. For these comparisons, we analyzed high-level beta diversity differences by using the 65 *C. elegans* population microbiome samples that were pre-processed and variance stabilized. The exact numbers of replicates remaining per treatment are reported in figure legends. All ANOSIMs were conducted with 999 permutations, and ANOSIM R statistics (R^2^).

For differential abundance of taxa analyses, we corrected for batch effects by incorporating batch as a term in the design formula of an ANCOM analysis [47]. Again, this test used the 65 pre-processed *C. elegans* microbiome samples, with exact n-values reported in the figure legend.

To analyze how transcript abundances related to *Enterococcus* abundance amongst the microbiome and initial *Enterococcus* accumulation in *C. elegans*, we calculated Pearson’s correlation coefficients. We also calculated Pearson’s correlation coefficient to analyze how *Enterococcus* abundance in the microbiome related to initial colonization and protection persistence. Since these comparisons were not within the same batch, but rather between batches from different experiments, we had to aggregate treatment samples and were limited to one data point per treatment.

To test for treatment differences in colonization and protection, we first analyzed data distributions and then used parametric or nonparametric tests where appropriate. We used one-tailed t-tests (with Holm corrected *p*-values) to test whether *E. faecalis* P accumulated more than other *E. faecalis* strains in nematode guts. To test for differences in symbiont-mediated protection against *S. aureus* across treatments, we used one-tailed Wilcoxon ranked sum tests (with Holm corrected p-values). To test whether there was a correlation between colonization and protection persistence, we calculated Pearson’s correlation coefficient.

## Supplementary information

**Additional file 1:Supplementary Fig. 1.** Alpha diversity measurements of *C. elegans* microbiota after compost exposure. Treatments are of different early exposures, prior to compost exposure. **a.** Observed ribosomal sequence variant measurement. **b.** Shannon diversity measurements. **c.** Chao 1 diversity measurement. Plotted with median (line), hinges as first and third quartiles (25th and 75th percentiles), and ends as ranges. Anc = *E. faecalis* ancestor. NP = *E. faecalis* no enhanced protection, *n* = 10; *E. faecalis* P = *E. faecalis* enhanced protection, n = 10 Pm = *P. mendocina*; n = 10; OP50 = *E. coli* OP50, *n* = 5. Full ANOVA tables in Supplementary Tables 1–4. **Supplementary Fig. 2.***E. faecalis* CFUs in *C. elegans* and relative abundance of *Enterococcus* amongst microbiome. X-axis is *C. elegans* gut bacterial colony-forming units (CFUs) after exposure to *E. faecalis* Anc, *E. faecalis* NP, or *E. faecalis* P. Y-axis is relative abundance of *Enterococcus* in *C. elegans* amongst microbiome. There was no significant correlation between *E. faecalis* CFUs and *Enterococcus* relative abundance. Error bars = ± s.e. Anc = *E. faecalis* ancestor. NP = *E. faecalis* no enhanced protection. *E. faecalis* P = *E. faecalis* enhanced protection. **Supplementary Fig. 3.** Relative abundance of *Enterococcus* in microbiome and proportion dead *C. elegans*. X-axis is proportion dead *C. elegans* after *S. aureus* exposure. Y-axis is relative abundance of *Enterococcus* in *C. elegans* amongst microbiome. There was no significant correlation between *E. faecalis* CFUs and *Enterococcus* relative abundance. Error bars = ± s.e. Anc = *E. faecalis* ancestor. NP = *E. faecalis* no enhanced protection. *E. faecalis* P = *E. faecalis* enhanced protection.

**Additional file 2:Supplementary Table 1.** Alpha diversity measurements of *C. elegans* microbiota after compost exposure. Treatments consist of the different symbionts colonizing nematodes prior to compost exposure. Anc = *E. faecalis* ancestor. NP = *E. faecalis* no enhanced protection. *E. faecalis* P = *E. faecalis* enhanced protection. **Supplementary Table 2.** ANOVA and Tukey-HSD tables for model for the effect of batch and treatment on observed RSVs. **Supplementary Table 3.** ANOVA and Tukey-HSD tables for model for the effect of batch and treatment on Chao 1 diversity. **Supplementary Table 4.** ANOVA table for model for the effect of batch and treatment on Shannon diversity.

## Data Availability

The packages and pipelines used are available, with documentation, on their respective sites and repositories. The main pipelines used, DADA2 (https://github.com/benjjneb/dada2), and phyloseq (https://joey711.github.io/phyloseq/) are all open-source and publicly available. R markdown files for implementing these packages on our data are available in supplementary files (Supplementary File 1). The 16S rRNA dataset supporting the conclusion of this article is available in the EMBL-EBI repository under the primary accession number PRJEB26987. Phenotypic data are in the supplementary materials.

## Abbreviations

ASVs: Amplicon sequence variants
ANOSIM: Analysis of Similarity
ANOVA: Analysis of Variance
*E.faecalis* P: *E.faecalis*, enhanced protection
*E.faecalis* NP: *E. faecalis*, no enhanced protection
*E.faecalis* Anc: Ancestral *E.faecalis*
