# Supplementary material for: Impacts of a novel defensive symbiosis on the nematode host microbiome
Source: BMC Microbiol. 2020 Jun 15;20:159. doi: 10.1186/s12866-020-01845-0 (PMC7296725; doi:10.1186/s12866-020-01845-0)
Supplement: Supplementary file 1 — Additional file 1:Supplementary Fig. 1. Alpha diversity measurements of C. elegans microbiota after compost exposure. Treatments are of different early exposures, prior to compost exposure. a. Observed ribosomal sequence variant measurement. b. Shannon diversity measurements. c. Chao 1 diversity measurement. Plotted with median (line), hinges as first and third quartiles (25th and 75th percentiles), and ends as ranges. Anc = E. faecalis ancestor. NP = E. faecalis no enhanced protection, n = 10; E. faecalis P = E. faecalis enhanced protection, n = 10 Pm = P. mendocina; n = 10; OP50 = E. coli OP50, n = 5. Full ANOVA tables in Supplementary Tables 1–4. Supplementary Fig. 2.E. faecalis CFUs in C. elegans and relative abundance of Enterococcus amongst microbiome. X-axis is C. elegans gut bacterial colony-forming units (CFUs) after exposure to E. faecalis Anc, E. faecalis NP, or E. faecalis P. Y-axis is relative abundance of Enterococcus in C. elegans amongst microbiome. There was no significant correlation between E. faecalis CFUs and Enterococcus relative abundance. Error bars = ± s.e. Anc = E. faecalis ancestor. NP = E. faecalis no enhanced protection. E. faecalis P = E. faecalis enhanced protection. Supplementary Fig. 3. Relative abundance of Enterococcus in microbiome and proportion dead C. elegans. X-axis is proportion dead C. elegans after S. aureus exposure. Y-axis is relative abundance of Enterococcus in C. elegans amongst microbiome. There was no significant correlation between E. faecalis CFUs and Enterococcus relative abundance. Error bars = ± s.e. Anc = E. faecalis ancestor. NP = E. faecalis no enhanced protection. E. faecalis P = E. faecalis enhanced protection. [file 12866_2020_1845_MOESM1_ESM.docx]

# Supplementary Figures

**
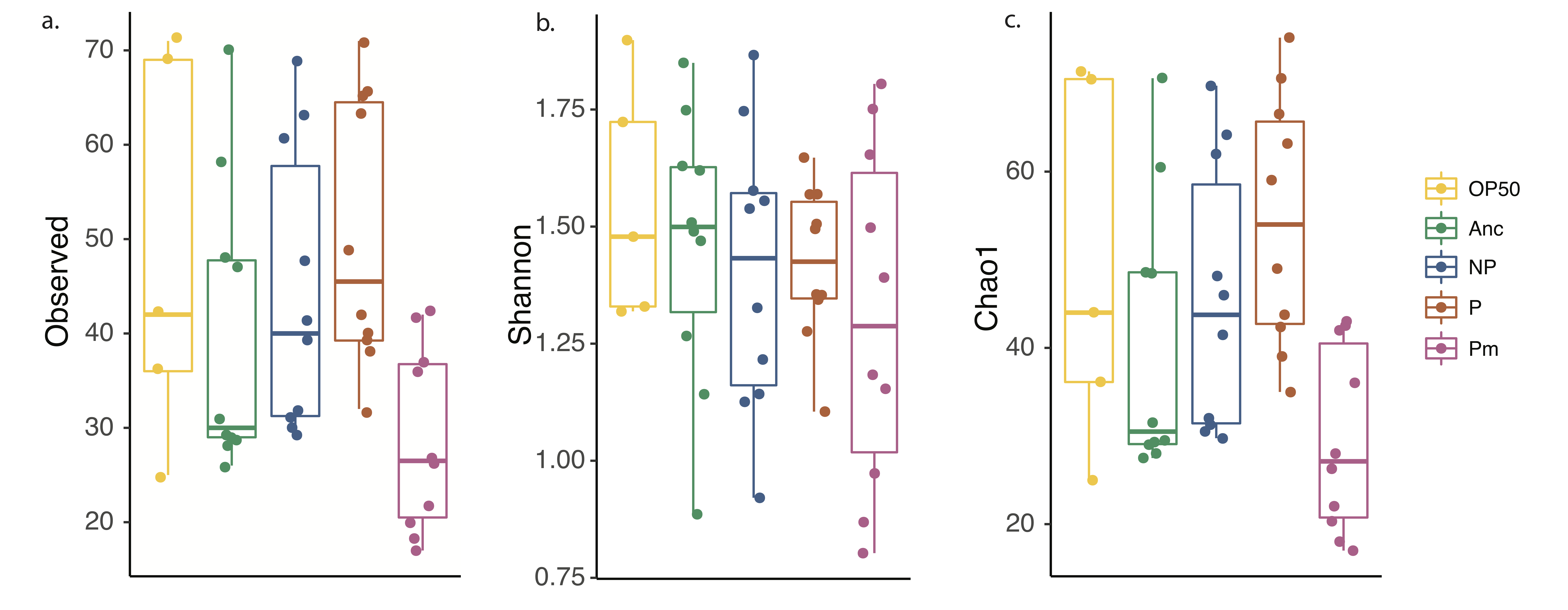
**

Supplementary Figure 1. Alpha diversity measurements of *C. elegans* microbiota after compost exposure. Treatments are of different early exposures, prior to compost exposure. **a.** Observed ribosomal sequence variant measurement. **b.** Shannon diversity measurements. **c.** Chao 1 diversity measurement. Plotted with median (line), hinges as first and third quartiles (25^th^ and 75^th^ percentiles), and ends as ranges. Anc = *E. faecalis* ancestor. NP = *E. faecalis* single-evolved, n = 10; *E. faecalis* P = *E. faecalis* co-colonized evolved, n = 10 Pm = *P. mendocina*; n = 10; OP50 = *E. coli* OP50, n = 5. Full ANOVA tables in Supplementary Tables 1-4.

**
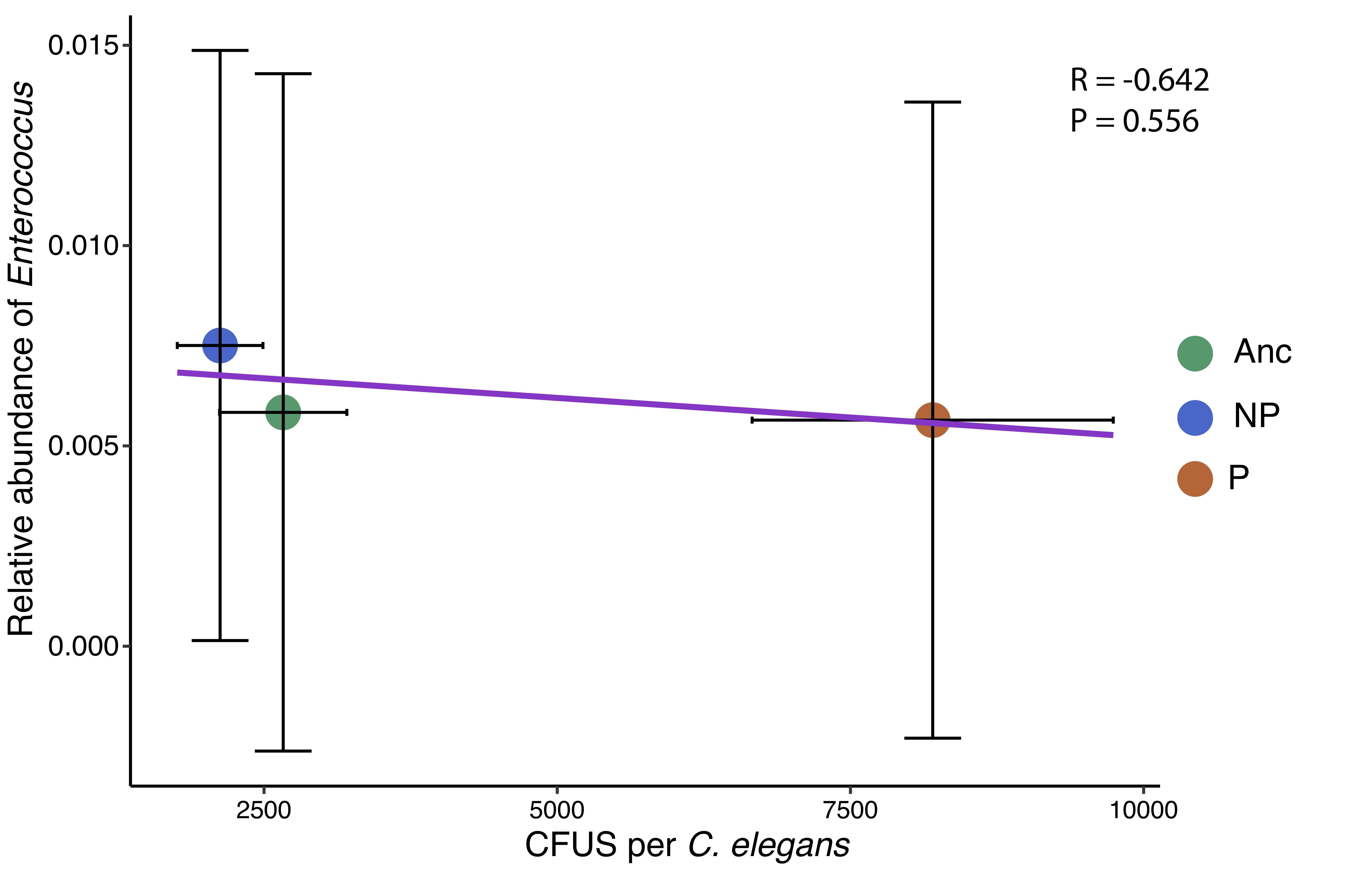
**

Supplementary Figure 2. *E. faecalis* CFUs in *C. elegans* and relative abundance of *Enterococcus* amongst microbiome. X-axis is *C. elegans* gut bacterial colony-forming units (CFUs) after exposure to *E. faecalis* Anc, *E. faecalis* NP, or *E. faecalis* P. Y-axis is relative abundance of *Enterococcus* in *C. elegans* amongst microbiome. There was no significant correlation between *E. faecalis* CFUs and *Enterococcus* relative abundance. Error bars = ± s.e. Anc = *E. faecalis* ancestor. NP = *E. faecalis* single-evolved. *E. faecalis* P = *E. faecalis* co-colonized evolved.

**
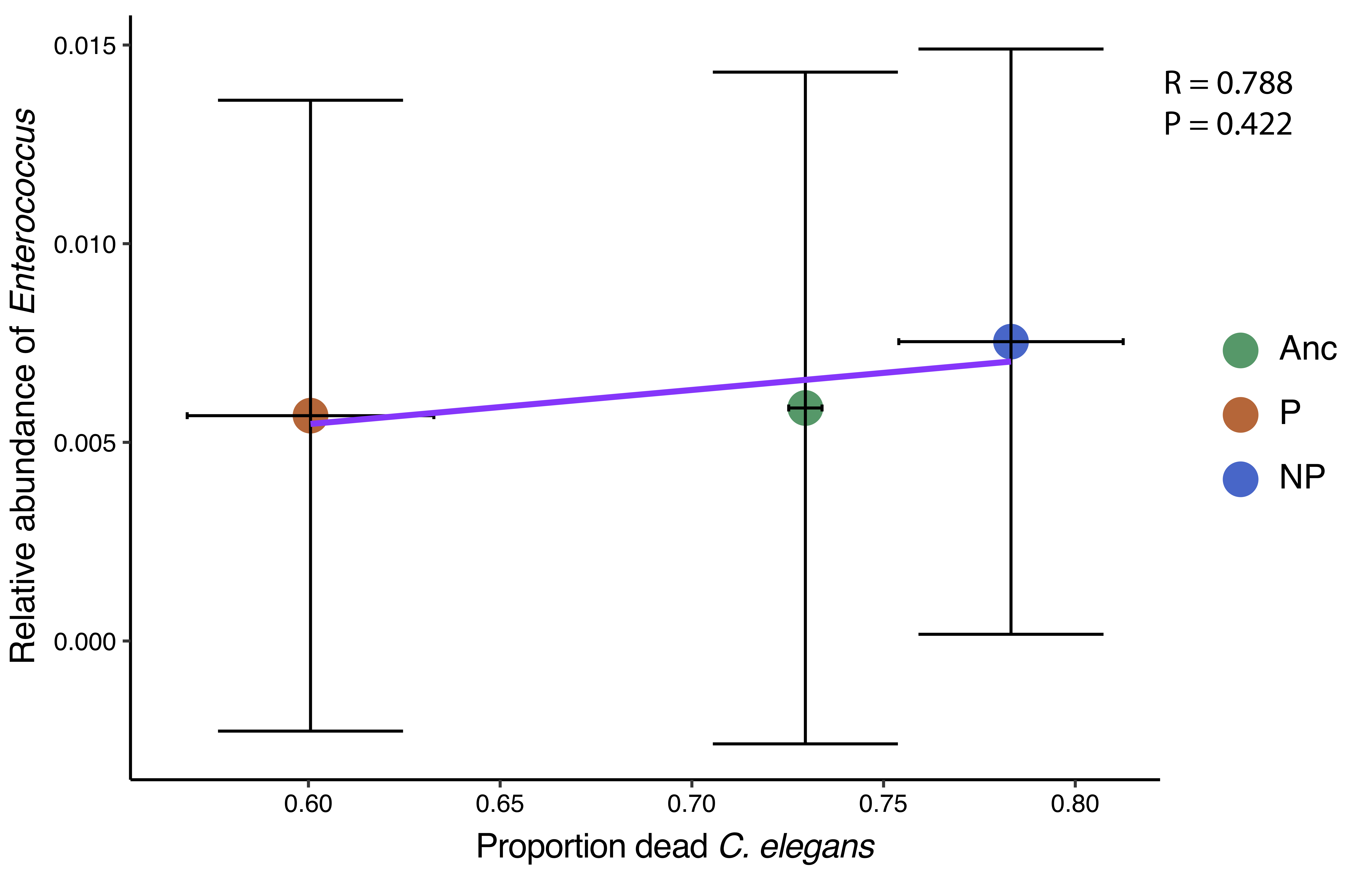
**

Supplementary Figure 3. Relative abundance of *Enterococcus* in microbiome and proportion dead *C. elegans*. X-axis is proportion dead *C. elegans* after *S. aureus* exposure. Y-axis is relative abundance of *Enterococcus* in *C. elegans* amongst microbiome. There was no significant correlation between *E. faecalis* CFUs and *Enterococcus* relative abundance. Error bars = ± s.e. Anc = *E. faecalis* ancestor. NP = *E. faecalis* single-evolved. *E. faecalis* P = *E. faecalis* co-colonized evolved.

##### 
