# Supplementary material for: Impacts of a novel defensive symbiosis on the nematode host microbiome
Source: BMC Microbiol. 2020 Jun 15;20:159. doi: 10.1186/s12866-020-01845-0 (PMC7296725; doi:10.1186/s12866-020-01845-0)
Supplement: Supplementary file 2 — Additional file 2:Supplementary Table 1. Alpha diversity measurements of C. elegans microbiota after compost exposure. Treatments consist of the different symbionts colonizing nematodes prior to compost exposure. Anc = E. faecalis ancestor. NP = E. faecalis no enhanced protection. E. faecalis P = E. faecalis enhanced protection. Supplementary Table 2. ANOVA and Tukey-HSD tables for model for the effect of batch and treatment on observed RSVs. Supplementary Table 3. ANOVA and Tukey-HSD tables for model for the effect of batch and treatment on Chao 1 diversity. Supplementary Table 4. ANOVA table for model for the effect of batch and treatment on Shannon diversity. [file 12866_2020_1845_MOESM2_ESM.docx]

# Supplementary Tables

Supplementary Table 1. Alpha diversity measurements of *C. elegans* microbiota after compost exposure. Treatments consist of the different symbionts colonizing nematodes prior to compost exposure. Anc = *E. faecalis* ancestor. NP = *E. faecalis* single-evolved. *E. faecalis* P = *E. faecalis* co-colonized evolved.

| *Treatment* | *Observed RSVs*  (Mean ± s.e.) | *Shannon* | *Chao 1* |
| --- | --- | --- | --- |
| *Anc* | 39.7 ± 5.00 | 1.47 ± 0.09 | 41.4 ± 5.57 |
| *NP* | 44.4 ± 4.56 | 1.40 ± 0.09 | 46.2 ± 4.84 |
| *P* | 50.0 ± 4.31 | 1.42 ± 0.05 | 51.5 ± 4.47 |
| *OP50* | 48.4 ± 8.99 | 1.55 ± 0.12 | 49.3 ± 8.93 |
| *Pm* | 28.4 ± 3.00 | 1.30 ± 0.12 | 28.6 ± 3.06 |

Supplementary Table 2. ANOVA and Tukey-HSD tables for model for the effect of batch and treatment on observed RSVs.

|  | DF | SS | MS | F | P |
| --- | --- | --- | --- | --- | --- |
| Batch | 1 | 283 | 283 | 1.37 | 0.249 |
| Treatment | 4 | 3182 | 795 | 3.84 | 0.010 |
| Residuals | 39 | 8077 | 207 |  |  |
|  |  |  |  |  |  |
|  |  | Tukey HSD |  |  |  |
|  | diff | lwr | upr | adj-P |  |
| P-Anc | 11.5 | -6.90 | 29.9 | 0.396 |  |
| OP50-Anc | 12.7 | -9.81 | 35.3 | 0.498 |  |
| Pm-Anc | -10.3 | -28.7 | 8.10 | 0.506 |  |
| NP-Anc | 5.6 | -12.8 | 24.0 | 0.906 |  |
| OP50-P | 1.23 | -21.3 | 23.8 | 1.00 |  |
| Pm-P | -21.8 | -40.2 | -3.40 | 0.013 |  |
| NP-P | -5.9 | -24.3 | 12.5 | 0.889 |  |
| Pm-OP50 | -23.0 | -45.6 | -0.485 | 0.043 |  |
| NP-OP50 | -7.13 | -29.7 | 15.4 | 0.894 |  |
| NP-Pm | 15.9 | -2.50 | 34.3 | 0.118 |  |

Supplementary Table 3. ANOVA and Tukey-HSD tables for model for the effect of batch and treatment on Chao 1 diversity.

|  | DF | SS | MS | F | P |
| --- | --- | --- | --- | --- | --- |
| Batch | 1 | 214 | 214 | 0.938 | 0.339 |
| Treatment | 4 | 3356 | 839 | 3.67 | 0.012 |
| Residuals | 39 | 8907 | 228 |  |  |
|  |  |  |  |  |  |
|  |  | Tukey HSD |  |  |  |
|  | diff | lwr | upr | adj-P |  |
| P-Anc | 11.9 | -7.40 | 31.3 | 0.408 |  |
| OP50-Anc | 13.5 | -10.2 | 37.2 | 0.487 |  |
| Pm-Anc | -10.3 | -29.6 | 9.02 | 0.553 |  |
| NP-Anc | 5.84 | -13.5 | 25.2 | 0.908 |  |
| OP50-P | 1.57 | -22.1 | 25.2 | 1.00 |  |
| Pm-P | -22.2 | -41.6 | -2.91 | 0.017 |  |
| NP-P | -6.09 | -25.4 | 13.2 | 0.895 |  |
| Pm-OP50 | -23.8 | -47.5 | -0.138 | 0.048 |  |
| NP-OP50 | -7.66 | -31.3 | 16.0 | 0.885 |  |
| NP-Pm | 16.1 | -3.18 | 35.5 | 0.140 |  |

Supplementary Table 4. ANOVA table for model for the effect of batch and treatment on Shannon diversity.

|  | DF | SS | MS | F | P |
| --- | --- | --- | --- | --- | --- |
| Batch | 1 | 0.412 | 0.412 | 5.484 | 0.024 |
| Treatment | 4 | 0.151 | 0.0379 | 0.504 | 0.733 |
| Residuals | 39 | 2.940 | 0.0751 |  |  |
